# Jumonji domain containing protein 6 (Jmjd6) modulates splicing and specifically interacts with arginine–serine-rich (RS) domains of SR- and SR-like proteins

**DOI:** 10.1093/nar/gku488

**Published:** 2014-06-09

**Authors:** Astrid Heim, Christina Grimm, Udo Müller, Simon Häußler, Mukram M. Mackeen, Juliane Merl, Stefanie M. Hauck, Benedikt M. Kessler, Christopher J. Schofield, Alexander Wolf, Angelika Böttger

**Affiliations:** 1 Department of Biology II, Ludwig Maximilians University, Munich, Großhaderner Strasse 2, 82152 Planegg-Martinsried, Germany; 2 Chemistry Research Laboratory and Oxford Centre for Integrative Systems Biology, University of Oxford, 12 Mansfield Road, Oxford OX1 3TA, UK; 3 School of Chemical Science, Faculty of Science and Technology, and Institute of Systems Biology (INBIOSIS) Universiti Kebangsaan Malaysia, 43600 Bangi, Selangor Darul Ehsan, Malaysia; 4 Research Unit Protein Science, Helmholtz Zentrum München-German Research Center for Environmental Health, Ingolstädter Landstrasse 1, 85764 Neuherberg, Germany; 5 Henry Wellcome Building for Molecular Physiology, Nuffield Department of Medicine, University of Oxford, Oxford OX3 7BN, UK; 6 Institute of Molecular Toxicology and Pharmacology, Helmholtz Zentrum München-German Research Center for Environmental Health, Ingolstädter Landstrasse 1, 85764 Neuherberg, Germany

## Abstract

The Fe(II) and 2-oxoglutarate dependent oxygenase Jmjd6 has been shown to hydroxylate lysine residues in the essential splice factor U2 auxiliary factor 65 kDa subunit (U2AF65) and to act as a modulator of alternative splicing. We describe further evidence for the role of Jmjd6 in the regulation of pre-mRNA processing including interactions of Jmjd6 with multiple arginine–serine-rich (RS)-domains of SR- and SR-related proteins including U2AF65, Luc7-like protein 3 (Luc7L3), SRSF11 and Acinus S′, but not with the bona fide RS-domain of SRSF1. The identified Jmjd6 target proteins are involved in different mRNA processing steps and play roles in exon dependent alternative splicing and exon definition. Moreover, we show that Jmjd6 modifies splicing of a constitutive splice reporter, binds RNA derived from the reporter plasmid and punctually co-localises with nascent RNA. We propose that Jmjd6 exerts its splice modulatory function by interacting with specific SR-related proteins during splicing in a RNA dependent manner.

## INTRODUCTION

Jumonji domain containing protein 6 (Jmjd6) is a member of the Fe(II) and 2-oxoglutarate (2OG) dependent oxygenase family (1). 2OG oxygenases couple the reaction of 2OG and oxygen with the two-electron oxidation of their primary substrates (proteins, lipids, nucleic acids or small molecules) with concomitant production of CO_2_ and succinate (2). The Jmjd6 protein is highly conserved throughout the animal kingdom and plays an important role in embryonic development. Jmjd6-knock-out experiments in vertebrates manifest serious developmental defects, e.g. in heart and brain and embryos died prenatally (3–5). In zebrafish developmental defects in addition to those involved in cardiovascular development, include those in somites and the notochord (6). Interestingly, loss of Jmjd6 function in the invertebrate *Caenorhabditis elegans* does not have any comparable phenotype (7).

Recently, Jmjd6 has been shown to interact with U2AF65, to catalyse hydroxylation of lysine residues in U2AF65 and to regulate alternative splicing (8). The biological importance of Jmjd6 in pre-mRNA splicing was demonstrated in mouse endothelial cells where Jmjd6 knockdown changed splicing of the vascular endothelial growth factor (VEGF)-receptor Flt1 pre-mRNA and thereby promoted expression of a soluble form of the receptor, which inhibits angiogenesis by binding to VEGF (9). More recently, the effect of iron on splicing of the pre-mRNA for ferrochelatase was demonstrated to work through regulation of the activity of Jmjd6 (10). However, Jmjd6 has never been identified in proteomic screens for components of the spliceosome (11–14) and is therefore probably not a constitutive part of it. Jmjd6 has also been reported to modify histones (15–17), the bromodomain containing protein Brd4 and the tumour suppressor p53 (18,19). Reported targets of the enzymatic activity of Jmjd6 include, on the one hand, lysine residues in histones, p53 and U2AF65 (8,19,20) and, on the other hand, methyl-arginine residues in histones and ERα (16,21). Thus, the detailed nuclear functions of Jmjd6, including the mechanisms by which it regulates splicing remain to be elucidated.

In the present study, we describe more detailed investigations on the interaction of Jmjd6 with splice factors. Jmjd6-pulldown assays revealed Jmjd6 interactions with a number of SR-like proteins, some of which were confirmed by co-immunoprecipitation experiments with endogenous Jmjd6. We show here that Jmjd6 binds the arginine–serine-rich (RS-) domains of U2AF65, Luc7L3, Acinus S′ and SRSF11. The interaction of Jmjd6 with the RS-domains of these target proteins is selective, because the RS-domain of SRSF1, for instance, is not bound within our limits of detection. The four analysed proteins are involved in different splicing and mRNA processing steps. Luc7L3 is the human homologue of yeast Luc7p, a component of the yeast U1 snRNP, which is involved in the U1 snRNP interaction with the nuclear cap binding complex (CBC) (22,23). Acinus is involved in splicing as part of the ASAP-complex, which contains SAP 18 (Sin 3 associated protein of 18 kDa) and RNPS1 and blocks the splicing activating function of RNPS1 (24). Acinus interacts with the core exon junction complex (EJC) (25). SRSF11 interacts with RNPS1, however, its function is not well characterised yet. In this study, we found that Jmjd6 formed a trimeric complex with the U2AF65/U2AF35 heterodimer. In accordance with these protein interactions of Jmjd6, we found that *jmjd6* knockdown stimulates splicing of a reporter gene and Jmjd6 overexpression inhibits it. In high resolution fluorescence microscopy Jmjd6 protein co-localises with nascent RNA in HeLa cells. Overall our results support the proposal that a major function of Jmjd6 is in splicing modulation and that this is achieved principally via its interaction with RS-domains of SR-like proteins.

## MATERIALS AND METHODS

### Cell culture, transfection and immunostaining

HeLa cells and human embryonic kidney (HEK) 293T cells were cultured in Dulbecco's modified Eagle's medium (DMEM) supplemented with 10% fetal calf serum, penicillin (100 U ml^−1^) and streptomycin (100 μg ml^−1^) at 37°C, 5% CO_2_. For microscopy HeLa cells were grown to 50–70% confluence on 18 × 18 glass coverslips and transfected with expression constructs using Lipofectamine 2000 (Invitrogen) according to the manufacturer's instructions. 24 hours post-transfection, cells were fixed with 4% paraformaldehyde (15 min at room temperature) and permeabilised with 1% Triton-X-100 in phosphate buffered saline (PBS). JMJD6 (ab10526, Abcam), anti-BrdU (B8434, Sigma), anti-HA (H6908, Sigma) and anti-U2AF65 (ab37483, Abcam) were used as primary antibodies, Cy3-coupled anti-mouse (Jackson Immuno Research), Alexa647 anti-mouse (Invitrogen) and Alexa488 anti-rabbit (Invitrogen) were used as secondary antibodies.

### Native gel analysis

The native gel analysis has been described recently (26). Briefly, lysates of HEK-293T cells were loaded on a non-denaturing native gel (7% acrylamide/bis-acrylamide (29:1), 80 mM Tris/HCl pH 7.3), blotted and subsequently stained with the appropriate antibodies. An Odyssey imager (LI-COR) has been used for antibody detection. Secondary antibodies were goat anti-mouse IRDye800 (926-32210) and goat anti-rabbit IRDye680 (926-32221). For RNase treatment RNase A has been added to the lysisbuffer (final concentration: 1 mg/ml) and incubated for 30 min.

### 3D-structured illumination microscopy

HeLa cells were grown to 60–70% confluency on 18 × 18 coverslips. The cells were treated with 20 mM 5-fluorouridine (5-FU) solved in DMEM for 3 min and then fixed 10 min with 2% formaldehyde. All washing steps after fixation were performed with 0.02% Tween in PBS (PBST). Cells were quenched in saturated glycin-solution and permeabilised with 0.5% Triton-X100 in PBS. Blocking was performed in 2% Bovine serum albumin (BSA) and 0,5% fish skin gelatin (FSG) in PBST. Primary antibodies used were: rabbit polyclonal anti JMJD6 (ab10526, Abcam), mouse monoclonal anti-BrdU (B8434, Sigma) and rabbit polyclonal anti-U2AF65 (ab37483, Abcam) Secondary antibodies (Jackson ImmunoResearch, Molecular Probes) were coupled to Alexa405 for blue fluorescence, Alexa488 for green fluorescence and Alexa594 for red fluorescence. Cells were post-fixed with 4% formaldehyde in PBS after incubation with secondary antibodies. After additional washing steps cells were counterstained with 200 ng/ml DAPI in PBST for 10 min. Cells were mounted on microscopy slides with Vectashield mounting medium (Vector Laboratories) and imaged using the DeltaVision OMX (Applied Precision).

### Co-immunoprecipitation experiments

HeLa cells were transiently transfected with C-terminal HA-tagged full-length Jmjd6 and green fluorescent protein (GFP)-tagged variants of either U2AF65, Acinus S′, SRSF11, Luc7L3 or SRSF1. The GFP-nanotrap (ChromoTek GmbH, Germany) has been used for immunoprecipitation as described previously (8). For RNase treatment RNase A has been added to the lysis buffer (final concentration: 1 mg/ml) and incubated for 30 min. Primary antibodies for western blotting were mouse anti-GFP antibody (11814460001, Roche) and rabbit anti-HA antibody (H6908, Sigma).

### In-gel-trypsin digestion and mass spectrometry of GFP-pulldown samples

The procedure used for analysis of proteins by mass spectrometry has been described previously (26).

### Immunoprecipitation of endogenous Jmjd6

For each immunoprecipitation experiment ca. 5 × 10^7^ HeLa cells were grown in DMEM (Biochrom) supplemented with 10% fetal calf serum, penicillin (100 U ml^−1^) and streptomycin (100 μg ml^−1^) at 37°C, 5% CO_2_. Cells were harvested, lysed for 30 min on ice in lysis buffer (150 mM NaCl, 10 mM Tris–HCl pH 7.5, 0.5% NP-40) and then sonicated for 20 s. After centrifugation, the supernatant was incubated at 4°C for 2 h with 5 μg of anti-Jmjd6 antibody (ab10526, Abcam) and for control with non-specific rabbit IgG (Merck Millipore). The lysate–antibody complexes were then added to 50 μl of Protein G Sepharose beads (4 fast flow, GE Healthcare) and incubated for 2 h at 4°C. Following the immunoprecipitation, beads were washed four times with wash buffer 1 (150 mM NaCl, 20mM Tris–HCl pH 7.5) and four times with wash buffer 2 (300 mM NaCl, 20 mM Tris–HCl pH 7.5) and then resuspended in Laemmli buffer.

### Label-free LC–MS/MS analysis of immunoprecipitation of endogenous Jmjd6

Each 10 μg of IP samples and controls were digested with a modified filter aided sample preparation (FASP) procedure (27). Briefly, the proteins were reduced and alkylated using dithiothreitol (DTT) and iodoacetamide (IAA) and then centrifuged through a 30 kDa cut-off filter device (PALL, Port Washington, USA), washed thrice with UA buffer (8 M urea in 0.1 M Tris/HCl pH 8.5) and twice with 50 mM ammonium bicarbonate. The proteins were digested on the filter for 2 h at room temperature using 1 μg Lys-C (Wako Chemicals, Neuss, Germany) and for 16 h at 37°C using 2 μg trypsin (Promega, Mannheim, Germany). The peptides were collected by centrifugation (10 min at 14 000 g), and the samples were acidified with 0.5% trifluoroacetic acid (TFA) and stored at −20°C. Before loading, the samples were centrifuged for 5 min at 4°C. LC–MS/MS analysis was performed as described previously (28). The samples were automatically injected and loaded onto the trap column at a flow rate of 30 μl/min in 3% buffer B (73% ACN/ 3% DMSO/ 0.1% formic acid (FA) in HPLC-grade water) and 97% buffer A (2% ACN/ 3% DMSO/0.1% FA in HPLC-grade water) (29). After 5 min, the peptides were eluted from the trap column and separated on the analytical column by a 140-min gradient from 3 to 35% of buffer B at 300 nl/min flow rate followed by a short gradient from 35 to 95% buffer B in 5 min. Between each sample, the gradient was set back to 3% buffer B and left to equilibrate for 20 min. From the MS prescan, the 10 most abundant peptide ions were selected for fragmentation in the linear ion trap if they exceeded an intensity of at least 200 counts and if they were at least doubly charged. During fragment analysis a high-resolution (60 000 full-width half maximum) MS spectrum was acquired in the Orbitrap with a mass range from 200 to 1500 Da.

The acquired spectra were loaded to the Progenesis LC–MS software (version 2.5, nonlinear) for label free quantification and analyzed as described previously (28,30). Briefly, profile data of the MS scans were transformed to peak lists with respective peak *m*/*z* values, intensities, abundances (areas under the peaks) and *m*/*z* width. MS/MS spectra were treated similarly. After reference selection, the retention times of the other samples were aligned by automatic alignment to an overlay of all features of at least 97%. Features with only one charge or more than seven charges were excluded from further analyses. All MS/MS spectra were exported as Mascot generic file and used for peptide identification with Mascot (version 2.4) in the Ensembl Human protein database (release 7 240 047 703 residues, 105 287 sequences). Search parameters used were: 10 ppm peptide mass tolerance and 0.6 Da fragment mass tolerance, one missed cleavage allowed, carbamidomethylation was set as fixed modification, methionine oxidation and asparagine or glutamine deamidation were allowed as variable modifications. A Mascot-integrated decoy database search calculated an average false discovery of <1% when searches were performed with a mascot percolator score cut-off of 15 and an appropriate significance threshold *P*. Peptide assignments were re-imported into the Progenesis LC–MS software and the abundances of all peptides allocated to each protein were summed up. The resulting normalized abundances were then used for calculation of enrichment factors of proteins.

### Double-reporter splicing assay

The plasmid pTN24, carrying the β-galactosidase (β-gal) and luciferase (luc) gene has been described elsewhere (31). HeLa cells have been transiently transfected with pTN24 and co-transfected with either empty pcDNA3 plasmid or full-length Jmjd6 in pcDNA3. β-gal and luc activity was detected in a luminometer (Lumat 9501, Berthold) 24 h post-transfection. Therefore, cells were lysed with passive lysis buffer of the dual-luciferase reporter assay system (Promega, Wisconsin, USA) for 15 min. Lysates were centrifuged at 14 000 g for 1 min. Luc activity was measured in 10 μl of the supernatant by using the dual-luciferase reporter assay system (Promega, Wisconsin, USA) and β-gal activity was analysed with the Galacto-Light system (Applied Biosystems) according to the manufacturer's instructions.

### Jmjd6 siRNA knock down experiments

Stealth siRNA-132 and siRNA-275 (Invitrogen) have been used to knock down Jmjd6 in HeLa cells. Jmjd6 siRNA-132 corresponds to nucleotides 132–156, siRNA-275 corresponds to nucleotides 275–299 of the Jmjd6-ORF. As a control experiment siRNA with no corresponding sequence in the JMJD6-ORF has been used (si-275-control). Mock-transfection without siRNA has been used as an additional control experiment.

HeLa cells were grown to 50–60% confluence in a 12-well and transfected with 200 nM siRNA (Lipofectamine 2000, Invitrogen) at day 1. At day 2 (24 h post-transfection) cells were passaged into a 6-well and transfected again with 200 nM siRNA. Efficient knock-down of Jmjd6 was measured at day 5 as monitored with anti-Jmjd6 western blots. β-Galactosidase and luciferase activity for the double-reporter splicing assay was analysed at day 5 (see above).

siRNA sequences:

siRNA-132: UAA CGU GGA AAG GGC AGA UGC UUU A

siRNA-275: GGA AAU AUC GGA ACC AGA AGU UCA A

siRNA-275-control: GGA UAU GGC CAA GAC GAA UUA ACA A

The nucleotide sequences of Jmjd6 and Jmjd6AxA in pcDNA3 plasmid were changed for transient expression in Jmjd6 knockdown cells (rescue experiment). The nucleotide changes are not altering the Jmjd6 amino acid sequence.

Nucleotide sequence Jmjd6 wildtype: 132-T AAC GTG GAA AGG GCA GAT GCT TTA-156

Nucleotide sequence Jmjd6 rescue: 132-C AAT GTA GAG AGA GCA GAT GCT TTA-156

### Reverse transcription/polymerase chain reaction (PCR) of reporter-gene mRNA

24 hours post-transfection mRNA was isolated from HeLa cells by using the Quickprep mRNA Purification Kit (GE Healthcare) and 100 ng of isolated mRNA were used for cDNA synthesis with the First-Strand cDNA Synthesis Kit (GE Healthcare) according to the manufacturer's instructions. PCR was carried out with primers GalFor and LucRev, which compass the intron region between β-galactosidase and luciferase in the reporter gene mRNA. The PCR products differ in size by 200 nucleotides due to occurrence of splicing (unspliced, spliced).

Primer-sequences:

GalFor: AAC ATC AGC CGC TAC AGT CAA

LucRev: ACG TGA TGT TCT CCT CGA TAT

### RNA-immunoprecipitation experiments

HEK 293T cells were transiently transfected with pTN24 (31) and pHA-Jmjd6 plasmids. The RNA-immunoprecipitation (RIP) was done using the EZ-Magna-RIP-Kit (Merck Millipore) according to manufacturer's instructions with either an anti-HA antibody or the corresponding rat-isotype control (ratIgG_1_, eBioscience, 16-4301-81 clone eBRG1). Contaminating DNA was digested prior to first strand cDNA-synthesis (GE Healthcare) using DNase I (RNase free, Roche). GalFor and LucRev primers (see above) were used for PCR amplification.

## RESULTS

### Jmjd6 co-precipitates with several proteins involved in mRNA processing

In a previous study, we identified the interaction of Jmjd6 with U2AF65 by tandem affinity purification (TAP-tag) coupled to mass spectrometry (LC–MS/MS) (8). In this study, we observed that additional splice related proteins interact with Jmjd6. We now carried out further interaction screens using GFP-pulldown experiments in HEK 293T cells (GFP-trap, (32)). We compared these results with the potential Jmjd6-binding proteins identified in our initial TAP-tag screen and in a recent pulldown-experiment carried out with HA-tagged Jmjd6 (33). We found 35 proteins appearing in at least three of these four independent pulldown experiments (Table 1). Most of them (22/35) were connected with pre-mRNA processing. Sixteen of the 22 splicing-associated proteins exhibited an arginine–serine-rich (RS) domain after Uniprot search and manual inspection (Table 1). Nine of these potential Jmjd6-target proteins had previously been shown to be present in the pre-spliceosomal A-complex (summarised in (34)). Except for SRSF11 none of the potential Jmjd6 targets belonged to the classical SR-protein family (35). We then confirmed some of these putative Jmjd6 target proteins by co-immunoprecipitation (co-IP) with endogenous Jmjd6 in HeLa cell lysates. Three independent co-IP experiments were carried out with anti-Jmjd6 antibody and the identity of co-precipitated proteins was analysed by label-free LC–MS/MS. Seven of the proteins listed in Table 1 were identified in all three experiments, including Luc7-like 2 and SRSF11 (Table 1 and Supplementary Figure S1).

**Table 1.** Jmjd6-interacting proteins with RS-domain

Accession number	Name	GFP-tag (IP 1)	GFP-tag (IP 2)	TAP-tag (Webby *et al*.)	HA-tag (Rahman *et al*.)	Endogenous interaction	RS domain (uniprot)
**Q9Y383**	**Putative RNA-binding protein Luc7-like 2**	+	+	+	+	+	+
**Q9NQ29**	**Putative RNA-binding protein Luc7-like 1**	+	+	+	+	+	+
**O95232**	**Luc7-like protein 3**	+	+	+	+	+	+
**Q16630**	**Cleavage and polyadenylation specificity factor subunit 6**	+	+	+	+	−	+
**Q01081**	**Splicing factor U2AF 35 kDa subunit**	+	+	+	+	−	+
**P26368**	**Splicing factor U2AF 65 kDa subunit**	+	+	+	+	+	+
**Q05519**	**Serine/arginine-rich splicing factor (SRSF) 11**	+	+	+	+	+	+
**Q14498**	**RNA-binding protein 39**	+	+	+	+	+	+
**Q7L4I2**	**Arginine/serine-rich coiled-coil protein 2**	+	+	−	+	−	+
**Q7L014**	**Probable ATP-dependent RNA helicase DDX46**	+	+	+	+	−	+
**Q8WXA9**	**Splicing regulatory glutamine/lysine-rich protein 1**	+	+	−	+	−	+
**Q9UQ35**	**Serine/arginine repetitive matrix protein 2**	+	+	−	+	−	+
**O75400**	**Pre-mRNA-processing factor 40 homolog A**	+	+	+	+	−	+
**Q9UKV3**	**Apoptotic chromatin condensation inducer in the nucleus (Acinus)**	+	+	+	−	−	+
**Q8N5F7**	**NF-kappa-B-activating protein**	+	+	+	+	−	+
**Q96IZ7**	**Serine/Arginine-related protein 53**	+	−	+	+	−	+
**O43809**	**Cleavage and polyadenylation specificity factor subunit 5**	+	+	−	+	−	−
**Q9UHX1**	**Poly(U)-binding-splicing factor PUF60**	+	+	+	+	+	−
**Q15637**	**Splicing factor 1**	+	+	−	+	−	−
**Q13435**	**Splicing factor 3B subunit 2**	+	+	−	+	−	−
**Q9UJV9**	**Probable ATP-dependent RNA helicase DDX41**	+	−	+	+	−	−
**P05455**	**Lupus La protein**	+	+	−	+	−	−
P11142	Heat shock cognate 71 kDa protein	+	+	−	+	−	−
P38646	Stress-70 protein, mitochondrial	+	+	−	+	−	−
P08107	Heat shock 70 kDa protein 1A/1B	+	+	−	+	−	−
P06748	Nucleophosmin	+	+	−	+	−	−
P50990	T-complex protein 1 subunit theta	+	+	−	+	−	−
P50991	T-complex protein 1 subunit delta	+	+	−	+	−	−
Q99832	T-complex protein 1 subunit eta	+	+	−	+	−	−
P10599	Thioredoxin	+	+	−	+	−	−
Q15084	Protein disulfide-isomerase A6	+	+	−	+	−	−
P62263	40S ribosomal protein S14	+	+	−	+	−	−
Q9BU76	Multiple myeloma tumor-associated protein 2	+	−	+	+	−	−
Q15365	Poly(rC)-binding protein 1	+	+	−	+	−	−
Q15366	Poly(rC)-binding protein 2	+	+	−	+	−	−

Summary of putative Jmjd6 binding proteins identified in two independent GFP-pulldown assays (IP 1 + 2) coupled to mass spectrometry (LC–MS/MS) after transient expression of Jmjd6-GFP in HEK 293T cells and comparison with recently published Jmjd6 pulldown results using TAP-tagged (8) or HA-tagged Jmjd6 in 293T cells (33). Only such proteins are listed that appeared in at least three of these four independent pulldown experiments. Column ‘endogenous interaction’ indicates Jmjd6–protein interactions that have been confirmed to occur on endogenous level, including this work and (8). Proteins connected to pre-mRNA processing are indicated in bold. Presence of RS-domains as defined by www.uniprot.com and as found by manual inspection is indicated in column ‘RS-domain’.

**Figure 1.**
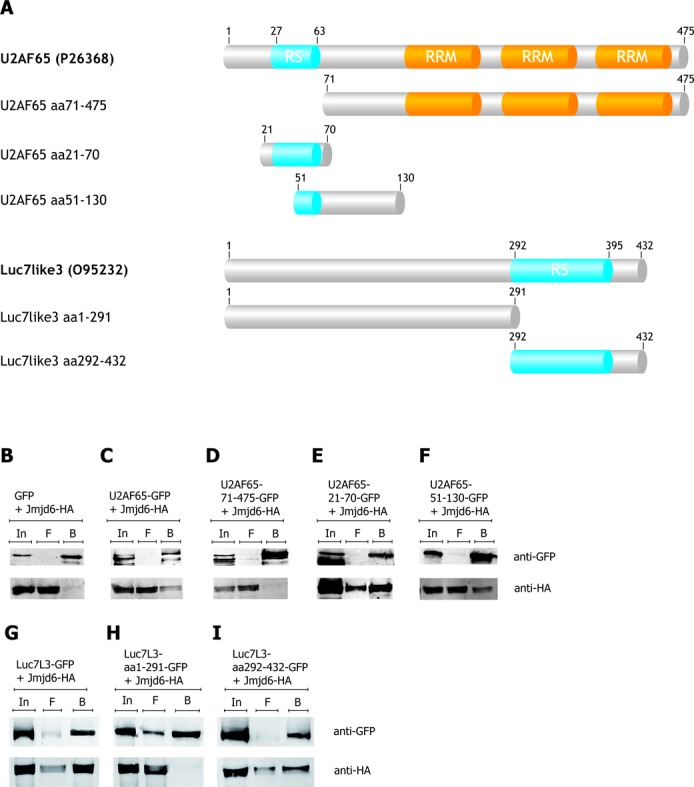
U2AF65 and Luc7L3 bind Jmjd6 via their RS domains. The U2AF65 protein exhibits two bona fide RNA binding domains (RRM) and a modified RRM, the so-called U2AF-homology motif (UHM), and an arginine–serine-rich (RS) domain (aa27–63). Various U2AF65 deletions and Luc7L3 variants were generated (**A**) and tested for Jmjd6 binding in co-immunoprecipitation assays (**B**–**F**). Full-length U2AF65 coupled to GFP and HA-tagged Jmjd6 were over-expressed in HEK 293T cells, lysed and immunoprecipitated with the GFP-nanotrap (32). Co-precipitation of HA-tagged Jmjd6 was tested in immunoblots with anti-HA antibody. Whereas the U2AF65-GFP co-precipitated Jmjd6-HA (**C**), the GFP-only control did not co-precipitate Jmjd6-HA (**B**). A U2AF65 lacking the RS-domain (U2AF65 71–465 GFP) did not interact with Jmjd6-HA (**D**). The RS-domain of U2AF65 (U2AF65 21–70 GFP) fused to GFP is sufficient to co-precipitate Jmjd6-HA (**E**). A truncated RS-domain (U2AF65 51–130 GFP) also interacted with Jmjd6-HA (**F**). A Luc7L3 variant lacking the RS-domain (Luc7L3 1–291 GFP) did not interact with Jmjd6-HA (**H**). But full-length Luc7L3-GFP (**G**) and the RS-domain of Luc7L3 fused to GFP (Luc7L3 292–432 GFP) are sufficient to co-precipitate Jmjd6-HA (**I**). In = input, F = flow-through, B = beads.

**Figure 2.**
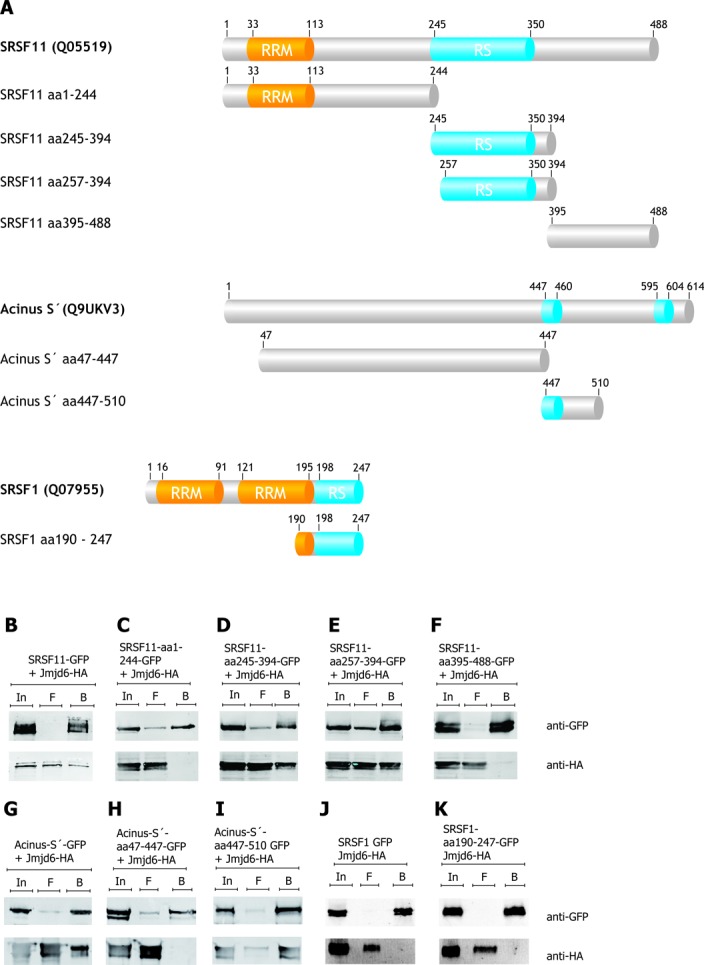
Acinus S′ and SRSF11, but not SRSF1 bind Jmjd6 via their RS domains. The domain structure of full-length Acinus S′, SRSF11, SRSF1 and deletions as investigated are shown schematically (**A**). GFP-tagged proteins were over-expressed with HA-tagged Jmjd6 in HEK 293T cells, lysed and immunoprecipitated with the GFP-trap (32). The SRSF11 protein fused to GFP co-precipitated Jmjd6-HA (**B**). The RS domain of SRSF11 is also sufficient to interact with Jmjd6-HA (**D**, **E**). SRSF11 variants lacking the RS domain did not interact with Jmjd6-HA (**C**, **F**). Acinus S′-GFP co-precipitated Jmjd6-HA (**G**), but not the truncated Acinus S′ version, lacking the RS domains (Acinus S′ 47–447 GFP) (**H**). The first RS domain (Acinus S′ 447–510 GFP) is sufficient to pull down Jmjd6-HA (**I**). The ‘classical’ SR–protein SRSF1 (37) exhibits an RS domain, but did not interact with Jmjd6-HA in our experiments (**J**). Fusing the RS-domain of SRSF1 to GFP also did not pull down Jmjd6-HA (**K**). In = input, F = flow-through, B = beads.

We have previously reported that peptides derived from RS-domains of U2AF65 and Luc7-like 2 were substrates for lysyl hydroxylation by Jmjd6. Moreover, we also found that lysyl residues outside the RS-domain of U2AF65 were hydroxylated by Jmjd6 *in vivo* and *in vitro* (8,36). We thus wanted to establish the interaction site(s) for Jmjd6 on other RS-domain containing proteins. We selected U2AF65, Luc7L3, Acinus S′ and SRSF11 for further analysis because they are involved in different RNA-processing steps (see ‘Introduction’ section). Figures 1 and 2 show schematics of the domain structures of these four SR-like proteins.

### Jmjd6 interacts with U2AF65, Luc7-like 3, SFSR11 and Acinus S′ via their RS domains

GFP-tagged U2AF65, Luc7L3, SRSF11 and Acinus S′ both in full-length and truncated versions were co-expressed with HA-tagged Jmjd6 in HEK293T cells (for schematic representation see Figures 1A and 2A). After cell-lysis, proteins were immunoprecipitated using the GFP-nanotrap method (32), separated by SDS-PAGE, immunoblotted and analysed with antibodies against GFP- or the HA-tag (Figures 1B–I and 2B–I). U2AF65-GFP was found to co-precipitate with Jmjd6-HA in such an experiment (Figure 1B and C); in contrast truncated U2AF65 lacking the RS domain did not bind to Jmjd6 (Figure 1D). However, the RS domain alone (aa21–70) coupled to GFP was sufficient for interaction with Jmjd6 (Figure 1E). Moreover, when we deleted parts of the RS domain of U2AF65 it still bound to Jmjd6 (Figure 1F). Co-immunoprecipitation experiments using Luc7L3-GFP, Acinus S′-GFP and SRSF11-GFP as well as several truncated versions of both proteins showed that they also did not interact with Jmjd6 in the absence of their RS domains and that an RS domain alone was sufficient for Jmjd6 binding (Figures 1G–I and 2B–I).

The specific interaction of Jmjd6 with RS-domains of different proteins poses the question why the classical SR–proteins (37) had not been enriched in any of the Jmjd6-interaction screens. We therefore analysed SRSF1 (Figure 2A). SRSF1-GFP pulldown in HEK293 cells did not co-precipitate HA-tagged Jmjd6 within limits of detection (Figure 2J). This was also true for the isolated RS-domain of SRSF1 fused to GFP (Figure 2K). Thus, the observed Jmjd6-RS-domain interactions exhibit specificity.

**Figure 3.**
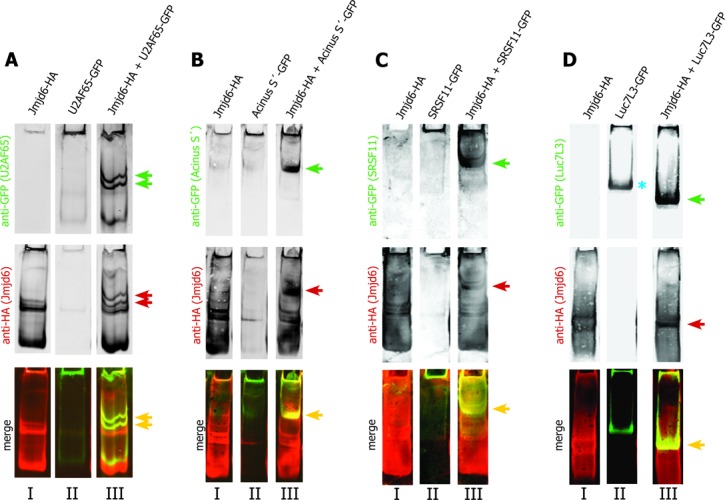
U2AF65, Acinus S′, SRSF11 and Luc7L3 interact with Jmjd6 oligomers. Jmjd6 assembles as oligomers in cells and these oligomers have been shown to run into native gels (26). We have tested whether Jmjd6 oligomers interact with substrate proteins by comparing the performance of proteins in native gels. Lysates of 293T cells expressing either HA-tagged Jmjd6 (lanes I) or substrate-GFP (U2AF65, Acinus S′, SRSF11, Luc7L3) (lanes II) or co-expressing both proteins (lanes III) were separated on native gels. Subsequently western blots were co-analysed with anti-HA antibody (Jmjd6) and anti-GFP antibody (green arrows) (U2AF65, Acinus S′, SRSF11, Luc7L3). Yellow bands in merged images indicate co-localisation of proteins in these bands (yellow arrows, red and green arrows indicate the same bands in single colour images). U2AF65-GFP did not run into the native gel significantly (**A**, II), but co-expression with Jmjd6-HA resulted in two prominent bands of U2AF65-GFP (green arrows in A, III), which co-localise with Jmjd6-HA bands (red arrows in A, III). Acinus S′-GFP did also not run into the native gel (**B**, II). In the presence of Jmjd6-HA it ran into the gel and co-migrated with Jmjd6 (green and red arrow in B, III). SRSF11-GFP did not run into the native gel significantly (**C**, II), but in the presence of Jmjd6-HA SRSF11-GFP co-migrated with Jmjd6 (green and red arrow in C, III). In contrast to U2AF65, Acinus S′ and SRSF11, Luc7L3 always ran into the gel in the absence of Jmjd6-HA (blue asterisk in **D**, II). Co-expression of Jmjd6-HA resulted in a prominent band representing a Luc7L3–Jmjd6 complex (green and red arrow in D, III).

The interaction of Jmjd6 with U2AF65, Acinus S′, Luc7L3 and SRSF11 was validated using native gel electrophoresis assays. Cells were co-transfected with plasmids encoding GFP-tagged versions of these proteins and HA-tagged Jmjd6. Lysates were then analysed using native Tris–borate–EDTA (TBE) gels. U2AF65-GFP did not run into the gel in significant amounts under these conditions. However, when Jmjd6 was co-expressed, both proteins migrated into the gel and were observed in two bands migrating slightly slower than Jmjd6 alone (Figure 3A). Acinus S′-GFP also did not migrate into the gel. When Jmjd6 was over-expressed, both proteins co-migrated as one strong band, migrating more slowly than Jmjd6 alone (Figure 3B). A similar result was obtained for SRSF11-GFP, which only marginally migrated into the gel alone. However, in the presence of Jmjd6, SRSF11-GFP was observed to migrate as a single strong band (Figure 3C). In contrast to Acinus S′ and SRSF11, Luc7L3-GFP always migrated into the gel by itself. Nevertheless, with Jmjd6 a faster migrating second Luc7L3 band co-localising with Jmjd6 (Figure 3D) was observed. These data confirmed that Jmjd6 interacts with the selected SR-like proteins.

**Figure 4.**
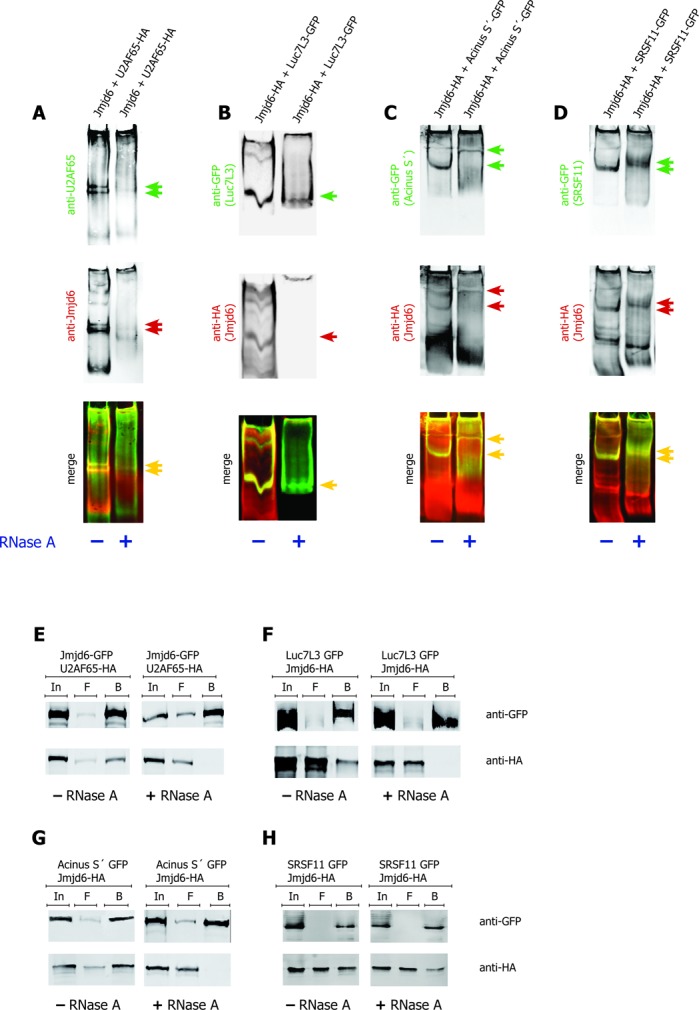
Interaction of Jmjd6 and SR–proteins is RNA dependent. The bands representing Jmjd6–SR–protein complexes in native gels disappear upon RNase A treatment. Both, Jmjd6 and U2AF65 were not found migrating into the gel after RNase treatment (**A**). Luc7L3 entered the gel after RNase A treatment, but was not able to recruit Jmjd6 (**B**). In case of Acinus S′ (**C**) and SRSF11 (**D**) the position of the Jmjd6–SR–protein complex was shifted upon RNase treatment. Complexes are indicated by arrows (A–D, red and green arrows indicate single colour bands that are co-localised as seen with yellow arrows in merged images). + = RNase A treatment; − = no RNase A treatment. RNase A treatment of 293T cell lysates from cells transfected with plasmids encoding GFP-tagged and HA-tagged proteins as indicated prior to anti-GFP pulldown, SDS-PAGE and western blot resulted in a breakdown of interaction of Jmjd6 with the SR–proteins U2AF65 (**E**), Luc7L3 (**F**) and Acinus S′ (**G**) but not with SRSF11 (**H**). In = input, F = flow-through, B = beads. Upper panels show precipitation of GFP-tagged proteins, lower panels show co-precipitation of HA-tagged proteins.

We then treated lysates with RNase before separating the proteins by native gel electrophoresis. In this case, migration of Jmjd6 into the gel was compromised and consequently, U2AF65 also did not migrate into the gel (Figure 4A). In samples over-expressing Luc7L3, the protein ran into the gel just as it did in the absence of over-expressed Jmjd6 (Figure 4B). However, when Jmjd6 was over-expressed together with Luc7L3, a Luc7L3–Jmjd6 complex was not observed after RNase treatment. In these experiments Jmjd6 failed to migrate into the gel after RNase treatment suggesting that Jmjd6 only moves towards the positively charged pole when it is bound to RNA. This is consistent with the basic character of Jmjd6 protein, which has an isoelectric point of 8.8. Luc7L3, which entered the gel after RNase treatment was not able to recruit Jmjd6 under these conditions (Figure 4B). In the presence of Acinus S′ and SRSF11 we observed that Jmjd6 had still migrated into the gel and formed a complex with the respective SR–protein, however, migration of the complexes was retarded. This may indicate that the oligomerisation state of Jmjd6 had changed (26). It is also possible that RNA was protected partially in Acinus–Jmjd6 and in SRSF11–Jmjd6 complexes (Figure 4C and D).

Previous work had revealed that the interaction of Jmjd6 with U2AF65 is abolished by treatment of the samples with RNase before precipitation (8). When samples with Luc7L3-GFP and Acinus S′-GFP were treated with 1 mg/ml RNase before GFP-pulldown, Jmjd6 was not co-precipitated (Figure 4E–G). In contrast, the interaction of Jmjd6 with SRSF11 was almost not affected by RNase (Figure 4H). These observations are consistent with our results from native gel electrophoresis. They imply that RNA is involved in targeting Jmjd6 to selected partner proteins.

### Jmjd6 co-localises with nascent RNA

**Figure 5.**
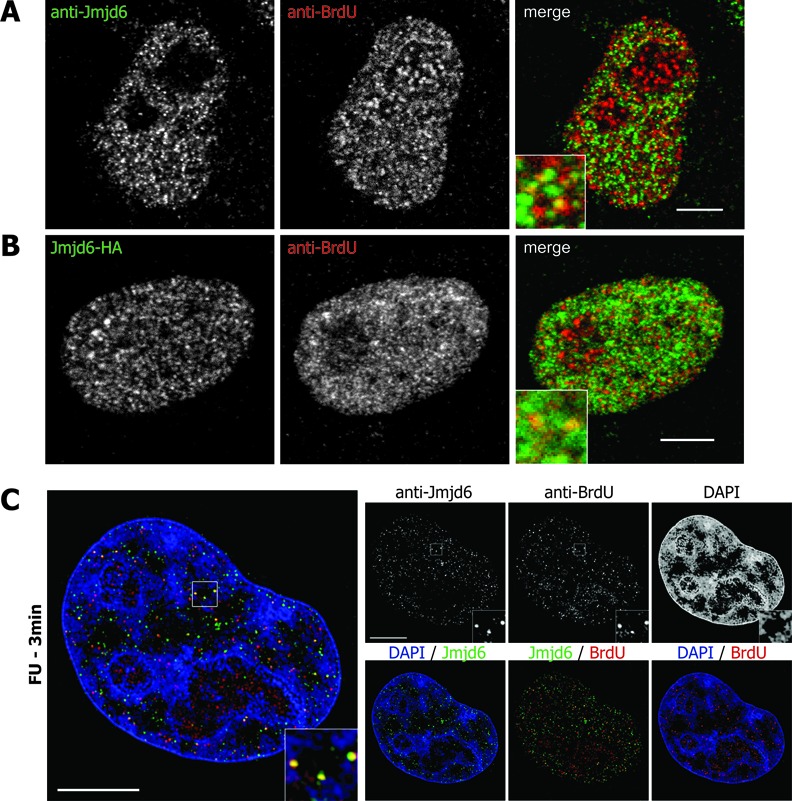
Jmjd6 co-localises with nascent RNA. HeLa cells were treated with 5-fluorouridine (5-FU) for 3 min and subsequently stained with anti-BrdU and anti-Jmjd6 antibody. Yellow dots indicate endogenous Jmjd6 (**A**) or overexpressed HA-tagged Jmjd6 (**B**) co-localising with nascent RNA in confocal microscopy. High-resolution microscopy and 3D SIM reconstruction (39) also revealed co-localisation of endogenous Jmjd6 with nascent RNA in individual nucleoplasmic spots. DNA is counterstained with DAPI. Central mid-sections of HeLa cell nuclei. Scale bars: 5 μm.

**Figure 6.**
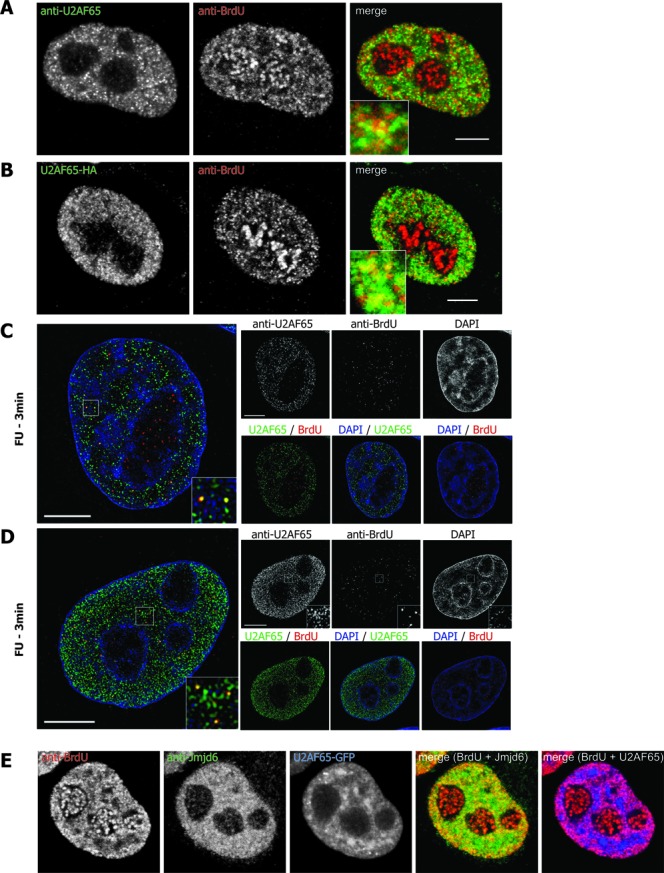
U2AF65 co-localises with nascent RNA. HeLa cells were treated with 5-fluorouridine (5-FU) for 3 min and subsequently stained with anti-BrdU and anti-Jmjd6 antibody. Discrete yellow dots indicate endogenous U2AF65 (**A** and **C**) or overexpressed HA-tagged U2AF65 (**B** and **D**) co-localising with nascent RNA in both confocal microscopy (**A** and **B**) and high-resolution microscopy and 3D SIM reconstruction (39) (C and D). Confocal cross-section showing GFP-U2AF65 over-expressing HeLa cells treated with 5-FU and stained with anti-Jmjd6 antibody (**E**). DNA is counterstained with DAPI. Central mid-sections of HeLa cell nuclei. Scale bars: 5 μm.

It had been reported previously that Jmjd6 can bind to RNA *in vitro* (38). We therefore investigated whether Jmjd6 co-localises with nascent RNA in cells. HeLa-cells were treated with FU for 3 min to label nascent RNA and FU-labelled RNA was subsequently detected with an anti-BrdU antibody. Cells were then co-stained with anti-Jmjd6 antibody. Laser confocal microscopy showed co-localisation of endogenous and overexpressed HA-tagged Jmjd6 with FU in several single dots (Figure 5A and B). High-resolution microscopy and 3D SIM (structured illumination microscopy) reconstruction (39) revealed co-localisation of endogenous Jmjd6 with nascent RNAs in very distinct dots in the nucleoplasm (Figure 5C). U2AF65 has been shown to be associated with mRNAs in several previous studies (40,41). In a control experiment endogenous and overexpressed HA-tagged U2AF65 also co-localises with nascent RNA in spots that are similar in appearance and number to the Jmjd6-co-localisation signals (Figure 6A and B). This was also shown in high-resolution microscopy and 3D SIM reconstruction (Figure 6C and D).

When we overexpressed U2AF65-GFP it was observed to partially localise to nuclear speckles. However, signals outside the speckles were largely found co-localised with nascent RNA. Endogenous Jmjd6 in such cells appeared to co-localise with nascent RNA very strongly too and also co-localised with U2AF65 (Figure 6E). These data indicate that Jmjd6 might associate with U2AF65-containing protein complexes on nascent RNA.

We therefore asked whether Jmjd6 was found in a complex with the U2AF65/U2AF35-dimer, which associates with the polypyrimidine tract and the AG-dinucleotide of the 3′ splice site during formation of the pre-initiation complex (42,43).

### Trimeric complex U2AF65/U2AF35/Jmjd6

**Figure 7.**
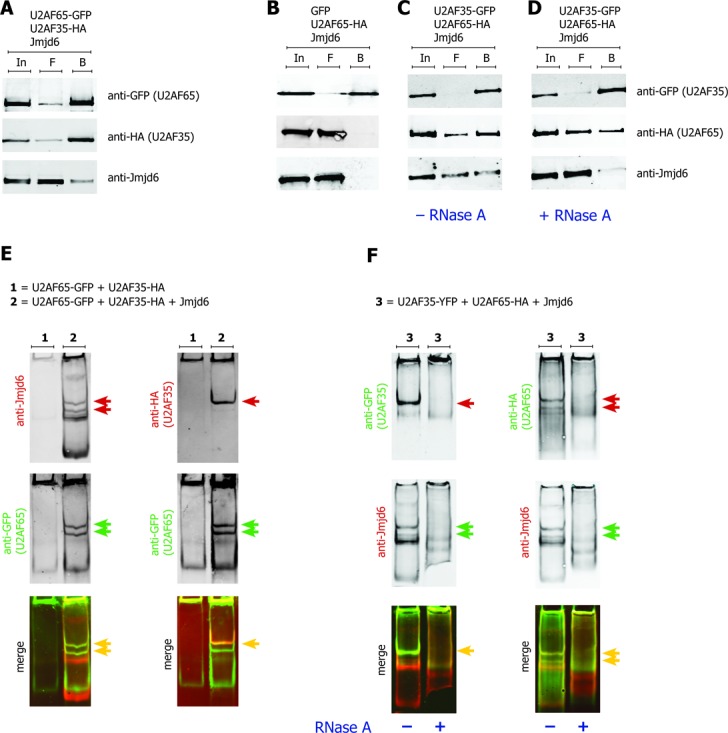
Jmjd6 forms a trimeric complex with U2AF65 and U2AF35 and RNA. GFP-tagged U2AF65, HA-tagged U2AF35 and Jmjd6 (pcDNA3) were co-expressed in 293T cells. The anti-GFP-pulldown of U2AF65-GFP resulted in co-precipitation of U2AF35 and Jmjd6 (**A**). Anti-GFP-pulldown of U2AF35-GFP resulted in co-precipitation of HA-tagged U2AF65 and Jmjd6 (**C**), a GFP-only control did not co-precipitate either of the two proteins (**B**). Treatment with RNase A prior to anti-GFP pulldown did not abolish the interaction of the U2AF dimer, but Jmjd6 disappeared from the complex (**D**). In = input, F = flow-through, B = beads. Lysates of 293T cells overexpressing U2AF65-GFP and U2AF35-HA were separated on a native gel and subsequently western blotted. Western blots were co-stained with indicated antibodies, yellow arrows indicate merged images. U2AF65-GFP and U2AF35-HA did not run into the native gel (**E**, 1). The co-expression of untagged Jmjd6 (pcDNA3) resulted in two distinct Jmjd6–U2AF65 complex bands (red and green arrows in E, left panel). U2AF35 was only present in one of these two bands (red and yellow arrow in E, right panel). Treatment with RNase A destroyed the Jmjd6–U2AF65–U2AF35 complex, as shown by disappearance of indicated bands in the RNase treated samples (**F**).

Figure 7A shows that U2AF65-GFP immunoprecipitates with both U2AF35 and Jmjd6, when all three proteins were over-expressed in HEK293T cells. Similarly, U2AF35-GFP precipitated both, U2AF65 and Jmjd6 (Figure 7B and C). When the samples were treated with RNase, U2AF65 was still precipitated with GFP-U2AF35, indicating that this interaction was not sensitive to RNase, which is in accordance with previously published data (44). However, Jmjd6 was not present in the complex after RNase treatment (Figure 7D). We then analysed the Jmjd6–U2AF65–U2AF35 complex on native TBE gels. When U2AF65 and U2AF35 were over-expressed they did not run into native TBE gels. When we additionally overexpressed Jmjd6 all three proteins appeared in one band, an additional band containing only Jmjd6 and U2AF65 was also seen (Figure 7E). All Jmjd6, U2AF65 and U2AF35 interaction was abolished after treatment of the samples with RNase (Figure 7F).

**Figure 8.**
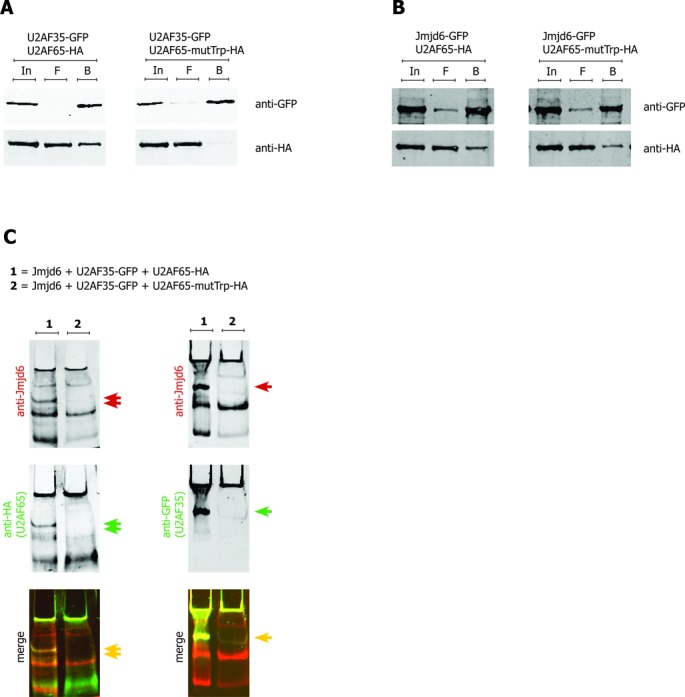
U2AF65–U2AF35 interaction is essential for Jmjd6 complex formation. Interaction of the U2AF-heterodimer is based on recognition of a specific tryptophane finger motif in U2AF65 by a modified RRM of U2AF35 (45). Mutation of the U2AF35 binding site in U2AF65 (U2AF65-mutTrp: W92A, P96G, P104G) inhibited the co-immunoprecipitation of HA-tagged U2AF65-mutTrp by GFP-tagged U2AF35 (**A**). In contrast, GFP-tagged Jmjd6 is able to pull down both, wildtype and mutTrp U2AF65 (**B**). Lysates of 293T cells overexpressing Jmjd6 (pcDNA3), U2AF35-GFP and either wildtype HA-tagged U2AF65 (lane 1) or HA-tagged U2AF65-mutTrp (lane 2) were separated on a native gel (**C**). As in Figure 7 two bands show co-migration of Jmjd6 with U2AF65 (red, green and yellow double arrows, left hand panel. (1) The slower migrating band contains U2AF35 (right hand panel, 1). With U2AF65-mutTrp the U2AF35-containing band is not present (both panels, (2) a trimeric U2AF65–U2AF35–Jmjd6 complex is not observed with U2AF65-mutTrp.

In order to analyse whether formation of the trimeric U2AF65/35/Jmjd6 complex is dependent on an intact U2AF65/U2AF35 interaction we mutated the U2AF65 interaction surface for U2AF35 by replacing the Trp-finger motif of U2AF65 (45) with alanine/glycine residues (U2AF65-mutTrp: W92A, P96G, P104G). In contrast to the interaction of U2AF35 and U2AF65-mutTrp, interaction of Jmjd6 with U2AF65-mutTrp was still observed in GFP-pulldown assays (Figure 8A and B). However, a trimeric complex was clearly not observed in native gel electrophoresis (Figure 8C). Thus, formation of a trimeric Jmjd6/U2AF65/U2AF35 complex is dependent on a sufficiently intact U2AF65/U2AF35 interaction.

### Jmjd6 inhibits splicing of a dual reporter gene

**Figure 9.**
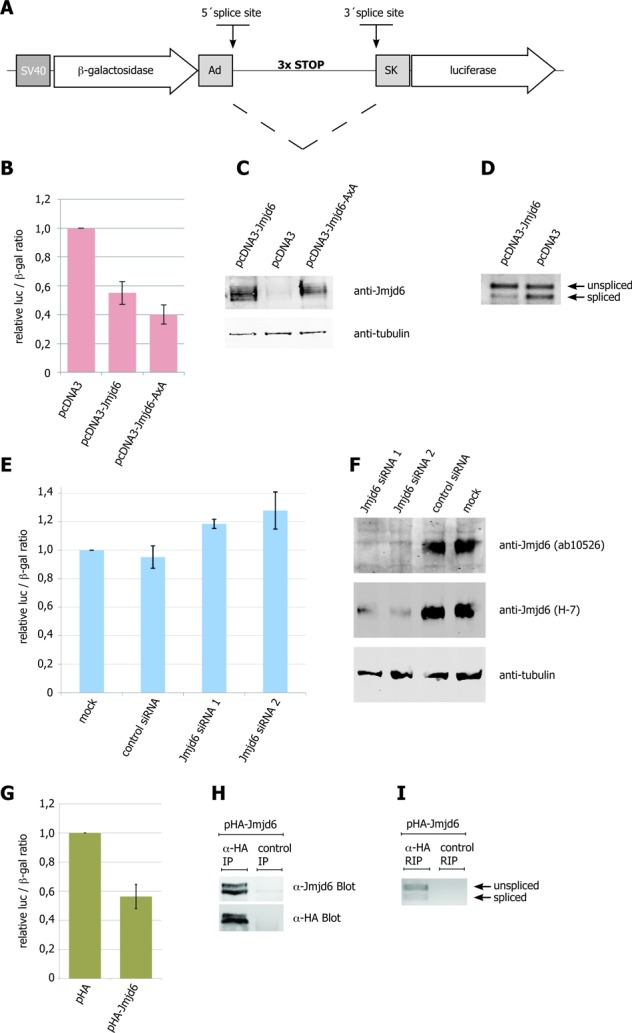
Jmjd6 abundance affects constitutive pre-mRNA splicing. The double-reporter splicing assay has been described previously (31). The pTN24 plasmid carries a β-galactosidase (β-gal) and a luciferase (luc) gene in frame, but separated by an intron. This intron encodes three translational stop codons (in-frame). The splice-site sequences are based on the genes encoding adenovirus (Ad) and the skeletal muscle isoform (SK) of human tropomyosin. Efficient splicing removes the translational stop codons and results in a fusion protein of both reporter genes, β-gal and luc. Plasmid is shown schematically in (**A**). Activities of luc and β-gal proteins have been measured after transient transfection of either pTN24 and pcDNA3-Jmjd6 or pTN24 and pcDNA3-Jmjd6-AxA or pTN24 and empty pcDNA3 plasmid in HeLa cells. Jmjd6 overexpression results in a decrease of splicing of the reporter mRNA, as seen in a decrease of luc activity (**B**). The same effect has been observed upon overexpression of a potentially enzymatic inactive Jmjd6 H187A&D189A variant (**B**). RT-PCR analysis confirmed the shift to an unspliced mRNA upon overexpression of Jmjd6 (**D**). Amounts of Jmjd6 protein in cells have been confirmed by western blot with anti-Jmjd6 antibody (**C**). siRNA-mediated knock-down of Jmjd6 increases splicing activity (**E**). Efficient knock-down of Jmjd6 protein has been detected by western blotting with anti-Jmjd6 antibody (**F**). Transient expression of HA-tagged Jmjd6 in HEK 293T cells resulted in a similar decrease of splicing of the reporter gene as observed with untagged Jmjd6 (**G**). HEK 293T cells transiently transfected with pTN24 and pHA-Jmjd6 were used for RNA-immunoprecipitation with either anti-HA or isotype specific control antibody. Specific immunoprecipitation of HA-tagged Jmjd6 was confirmed in western blots with anti-Jmjd6 or anti-HA antibody (**H**). Co-immunoprecipitation of reporter gene mRNA (spliced and unspliced) has been shown by RT-PCR (**I**). Error bars represent standard deviation of three independent experiments.

Given the interaction of Jmjd6 with the U2AF65/U2AF35 dimer and its co-localisation with nascent RNA we next analysed whether constitutive splicing was sensitive to the protein level of Jmjd6 in a cell using a constitutive dual reporter assay (31). The reporter plasmids encode two enzymes, β-galactosidase and luciferase. Their coding sequences are separated by an intron exhibiting three stop-codons in frame with the β-galactosidase coding sequence (Figure 9A). If this intron is spliced, both enzymes are expressed and their activities can be measured. In the absence of splicing, however, only β-galactosidase is expressed. The reporter mRNA was spliced much less efficiently, when Jmjd6 was over-expressed in HeLa cells. This was seen by the lower ratio of luciferase to β-galactosidase activity (Figure 9B and D). Expression of a Jmjd6 double mutation with H187 and D189 changed to alanine, which is catalytically inactive *in vitro* (8), had the same effect. On the other hand, Jmjd6 knockdown using siRNAs showed the opposite effect (Figure 9E and F). Expression of either Jmjd6 or the catalytically inactive Jmjd6 variant in Jmjd6 knockdown cells in a rescue experiment again reduced splice efficiency, indicating that Jmjd6 mediated splice inhibition was independent of its catalytic activity (Supplementary Figure S2). We confirmed that we were looking at splice inhibition by Jmjd6 by RT-PCR for the spliced and unspliced transcripts. The PCR products we obtained reflected the ratio of spliced to not spliced RNA that was indicated by comparison of the enzyme activities (Figure 9D). We also controlled the expression levels of Jmjd6 after over-expression and knockdown by SDS-PAGE and western blotting (Figure 9C and F). This indicated that Jmjd6 mediated splice inhibition of the reporter gene. We therefore investigated whether HA-tagged Jmjd6 bound to the plasmid derived mRNA. HA-tagged Jmjd6 showed the same effect on splicing as untagged Jmjd6 (Figure 9B and G). We found that the anti-HA antibody, but not the isotype specific control antibody, immunoprecipitated HA-tagged Jmjd6 (Figure 9H) and co-immunoprecipitated both, spliced and unspliced plasmid derived RNA (Figure 9I). This demonstrates that HA-Jmjd6 interacts with mRNA, either directly or indirectly.

## DISCUSSION

In this study, we have sought to understand the underlying molecular interactions involved in the splice modulatory function of Jmjd6. We found, that Jmjd6 interacts with multiple splice proteins, many of which have RS-domains. However, a closer analysis revealed that (with one exception for SRSF11) these do not include the so-called ‘classical’ SR–proteins, which have recently been defined as having one or two RNA binding motifs (RRMs) and a C-terminal RS-domain with a length of at least 50 amino acids, 40% of which should be RS or SR repeats (37). Instead, we find a subset of SR-like proteins with lesser numbers of RS-dipeptides, partially C-terminally located and also varying RNA-binding domains interacting with Jmjd6.

We carefully studied the interaction of Jmjd6 with Luc7L3, Acinus S′, SRSF11 and U2AF65; the results reveal that the presence of the RS-domains is essential for Jmjd6 interactions. In contrast, the classical SR–protein SRSF1 did not detectably interact with Jmjd6 in cells as was also observed for its isolated RS-domain. The RS-domain of SRSF1 comprises 50 residues with 14 repeats of an RS or SR dipeptide sequence. The RS-dipeptide content of the RS-domains of Acinus S′, Luc7L3 and U2AF65 is considerably lower than that of SRSF1, ranging from 20 to 32% (Supplementary Figure S3). An incomplete RS-domain of U2AF65, which also bound to Jmjd6 only contained 10 RS-repeats over a length of 50 amino acids. Thus, although clusters of RS-dipeptides comprise a common feature of Jmjd6 interaction domains, these alone are not sufficient. Overall these results imply that there is selectivity in terms of the RS-domains targeted by Jmjd6 though it is important to note that the selectivity may be context dependent.

Previously reported data have shown that Jmjd6 binds to RNA *in vitro* (38). In our experiments, the interaction of Jmjd6 with SR-like proteins was sensitive to RNase treatment to varying degrees. After treatment with RNase uncomplexed Jmjd6 no longer migrated into native TBE gels, indicating that the Jmjd6 oligomers we have identified on these gels before (26), are probably bound to RNA.

How can these data shed light on the function of Jmjd6 during splicing? In high-resolution microscopy we observed that Jmjd6 co-localises with nascent RNA in cells. U2AF65 is associated with pre-mRNA co-transcriptionally (40) and the co-localisation with nascent RNA that we observe very likely reflects this. Moreover, we also showed that Jmjd6 engages in a trimeric complex with the U2AF-dimer (U2AF65 and U2AF35). This was not simply caused by its interaction with RS-domains of U2AF65 and U2AF35, because the complex was dependent on an intact interaction of both U2AF subunits, which is mediated by contact of the 35 kDa subunit homology domain on the small subunit with a Trp-finger motif on the 65 kDa subunit (44).

Considering the overall results, the most likely scenario is that the role of Jmjd6 in modelling splicing includes its interaction with pre-mRNA while it interacts with the RS-domains of SR–proteins, thus modulating the splicing process. The sequence of Jmjd6 binding to nascent RNA or SR–proteins is presently unclear. Jmjd6 may be targeted by specific interactions with SR-like proteins, thus be recruited to the nascent RNA. Alternatively, Jmjd6 may recognise certain RNA sequences targeting it to splice sites, where it recruits RS-domain containing SR–proteins. Alternatively, a combination of both may be possible. However, Jmjd6 modulates pre-mRNA splicing and intron removal is affected, as clearly observed with constitutive splice reporter analyses. Jmjd6 overexpression was observed to decrease splicing of the reporter when Jmjd6 knockdown increased it. Moreover, Jmjd6 was co-precipitated with mRNA derived from transcription of the reporter plasmid. These data are in support of previous studies implying a role for Jmjd6 in pre-mRNA splicing (8–10,46).

The interaction of Jmjd6 with multiple SR–proteins, at least sometimes in an RNA dependent manner leads to the proposal that it acts as a facilitator protein for the fine-tuning of SR–protein functions. This role is likely to be highly context dependent and regulated by other multiple factors, including the Jmjd6 oligomerisation state. Jmjd6 forms large oligomers with a ring or a fibrillar structure depending on the presence of a serine-rich (polyS)-domain. Whilst wildtype Jmjd6 is localised mainly in the nucleoplasm, polyS-domain deleted versions of Jmjd6 are found in nuclear speckles and in the nucleolus (26).

SR–proteins function in many nuclear processes including splicing and transcriptional regulation (47). They have also been implicated in the process of transcriptional pausing of RNA polymerase II, where Jmjd6 has recently been shown to play a role in regulating promoter-proximal pause release (18,48).

Jmjd6 is most closely related to the enzyme factor inhibiting hypoxia inducible factor (FIH), which catalyses the hydroxylation of an asparagine residue in the C-terminal transcriptional activation domain of hypoxia inducible factor (HIF) alpha isoform (49,50); a modification that reduces the transcriptional activity of HIF in a proposed oxygen sensing mechanism, by preventing its interaction with 300 kDa coactivator protein (p300) (51). In addition to HIF-alpha FIH also accepts multiple other hydroxylation substrates of the ankyrin repeat domain containing protein family (52). It is proposed that the extent of the catalysed ankyrin hydroxylation regulates the amount of FIH available to hydroxylate HIF-alpha (53). Moreover, hydroxylation of ankyrin repeats has been shown to enhance their stability and this could be important for the function of this protein–protein interaction domain (54).

Jmjd6 was shown to hydroxylate specific lysine residues outside the RS-domain in U2AF65. At the same time it very efficiently hydroxylates RS-domain derived peptides (8), which makes it likely that the RS-domains, which bind to Jmjd6 are also hydroxylated on lysine residues. However, splice inhibition in our experiments seemed to be independent of its enzymatic activity, and therefore Jmjd6-binding of RS-domains may be sufficient for splice inhibition of the reporter gene.

In conclusion, we suggest that Jmjd6 interacts with RS-domains of specific SR-like proteins in a context dependent manner and thus participates in RNA-protein complexes, which form during splicing. In agreement with this Jmjd6 has an effect on splicing of a reporter gene. The function of the enzymatic dioxygenase activity of Jmjd6 in this context is presently not clear. Possible targets for hydroxylation include SR-like proteins, RNA and/or methylated RNA. Moreover, Jmjd6 autohydroxylation could be involved (8,18,54). This problem is more difficult to investigate than for FIH because Jmjd6 engages in multi-protein–RNA complexes.

## SUPPLEMENTARY DATA

Supplementary Data are available at NAR Online.

SUPPORTING INFORMATION
