# Supplementary material for: Jumonji domain containing protein 6 (Jmjd6) modulates splicing and specifically interacts with arginine–serine-rich (RS) domains of SR- and SR-like proteins
Source: Nucleic Acids Res. 2014 Jun 9;42(12):7833–50. doi: 10.1093/nar/gku488 (PMC4081092; doi:10.1093/nar/gku488)
Supplement: SUPPORTING INFORMATION [file supp_42_12_7833__index.html]

Jumonji domain containing protein 6 (Jmjd6) modulates splicing and specifically interacts with arginine–serine-rich (RS) domains of SR- and SR-like proteins — SUPPORTING INFORMATION 

# Jumonji domain containing protein 6 (Jmjd6) modulates splicing and specifically interacts with arginine–serine-rich (RS) domains of SR- and SR-like proteins

## SUPPORTING INFORMATION

**Files in this Data Supplement:**

- Supplemental Figures
