# Supplementary material for: Jumonji domain containing protein 6 (Jmjd6) modulates splicing and specifically interacts with arginine–serine-rich (RS) domains of SR- and SR-like proteins
Source: Nucleic Acids Res. 2014 Jun 9;42(12):7833–50. doi: 10.1093/nar/gku488 (PMC4081092; doi:10.1093/nar/gku488)
Supplement: SUPPORTING INFORMATION [file supp_gku488_nar-02988-a-2013-File012.pdf]

figure S1

A

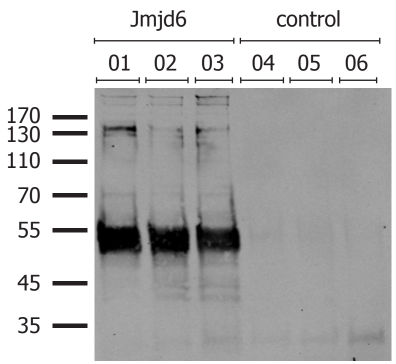

B

acc no	Unique peptides	Mascot percolator score	p-value	gene name	Enrichment factor Jmjd6-IPs/control
Q6NYC1	16	932	0.00000	Jumonji domain containing protein 6	269.45
Q9NQ29	1	127	0.00188	Putative RNA-binding protein Luc7-like 1	28.74
Q9Y383	5	283	0.00009	Putative RNA-binding protein Luc7-like 2	7.59
Q14498	2	104	0.00006	RNA-binding protein 39	5.25
Q05519	1	25	0.00231	Serine/arginine-rich splicing factor 11	4.73
O95232	2	130	0.00009	Luc7-like protein 3	4.02
Q9UHX1	4	131	0.00396	Poly(U)-binding-splicing factor PUF60	2.46

Figure S1: Immunoprecipitation experiments of endogenous Jmjd6 from HeLa cells. (**A**) Western blot stained with anti-Jmjd6 antibody (sc28348, Santa Cruz) showing the final beads fractions of three independent anti-Jmjd6 pulldown experiments (01-03) and three independent corresponding control (Merck Millipore) experiments (04-06). (**B**) Anti-Jmjd6 and control samples have been analysed by label-free LC-MS/MS analysis and abbundance of co-immunoprecipitated proteins has been analysed. Table indicates enrichment factor of Jmjd6 and co-immuno-precipitated SR-proteins in anti-Jmjd6 samples in comparison to control samples. Mass spectrometry analyses summarise the protein identifications of the samples 01-06 shown in (A).

figure S2

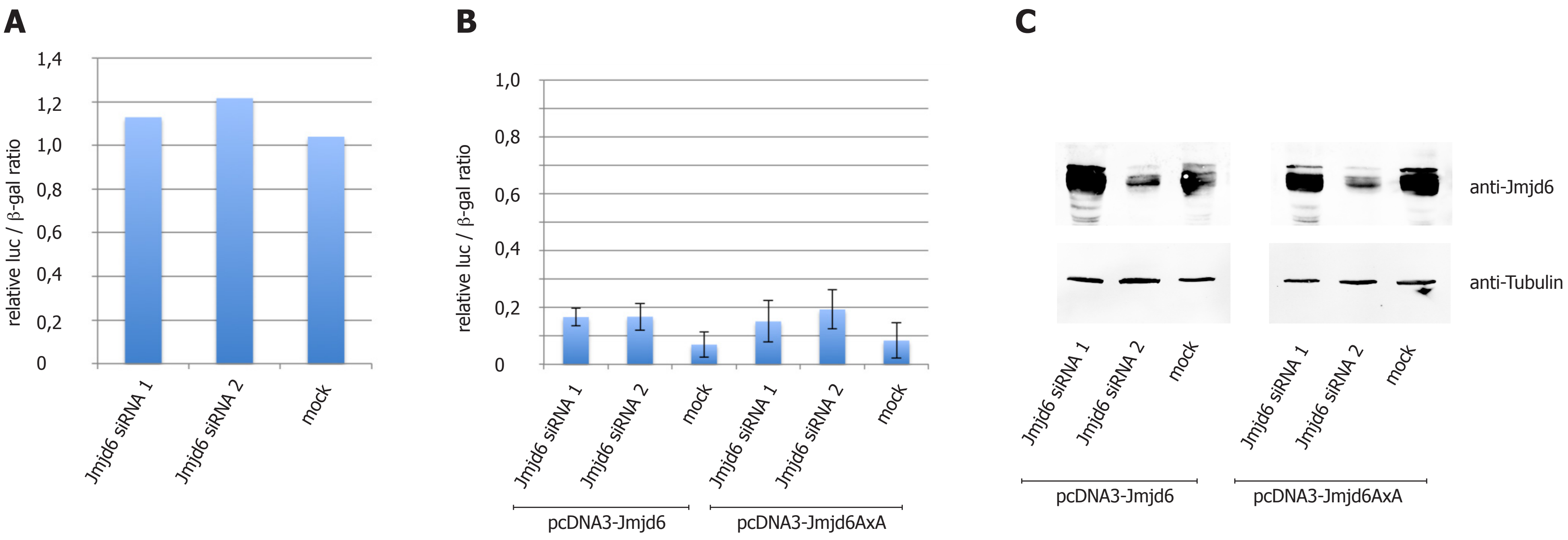

Figure S2: Rescue experiments after Jmjd6 knockdown in HeLa cells. siRNA-mediated knockdown of endogenous Jmjd6 increases splicing activity (**A) + (Fig. 9)**. A subsequent transient expression of wildtype Jmjd6 in the Jmjd6 knock down cells reduces splicing activity in the double-reporter splicing assay. A similar effect was observed for an enzymatically inactive Jmjd6AxA variant (**B**). Nucleotide sequence of the pcDNA3-Jmjd6 and pcDNA3-Jmjd6AxA constructs has been changed in order to avoid binding by siRNA1. Nucleotide changes did not result in a change in amino acid sequence (see material & methods). Jmjd6 levels were detected in immuno blots (**C**).

figure S3

A

SRSF11 (245-394):
RRHSRSRSRSRRRRTPSSSRHRRSRSRSRRRSHSKSRSRRRSKSPRRRRSHSRERGRRSRSTSKTRDKKKEDKEKKRSKTPPKSYSTARRSRSASRERRRRRSRSGTRSPKKPRSPKRKLSRSPSPRRHKKEKKKDKDKERSRDERERS

Luc7like3 (292-432):
RARDRERRKRSRSRSRHSSRTSDRRCSRSRDHKRSRSRERRRSRSRDRRRSRSHDRSERKHRSRSRDRRRSKSRDRKSYKHRSKSRDREQDRKSKEKEKRGSDDKKSSVKSGSREKQSEDTNTESKESDTKNEVNGTSEDIKSEGDTQSN

U2AF65 (21-70):
ENRHRKRSHSRSRSRDRKRRSRSRDRRNRDQRSASRDRRRRSKPLTRGA

Acinus S´(447-510):
PRSRSRSRDRRRKERAKSKEKKSEKKEKAQEEPPAKLLDDLFRKTKAAPCIYWLPLTDSQIVQK

B

SRSF1 (198-247):
RSPSYGRSRSRSRSRSRSRSRSNSRSRSYSPRRSRGSPRYSPRHSRSRSRT

Figure S3: Jmjd6 interacts with RS-domains of several SR-like proteins. Amino acid sequences of the Jmjd6-interacting domains of SRSF11, Luc7like3, U2AF65 and Acinus S´ as validated in co-immunoprecipitation (co-IP) experiments (**A**). The RS-domain of SRSF1 is not bound by Jmjd6 in these co-IP experiments (**B**).
